# Risk Factors Associated with Azotemia in Dogs Presented to the Chiang Mai University Veterinary Teaching Hospital, Thailand: A Retrospective Study (2017–2021)

**DOI:** 10.3390/ani15223313

**Published:** 2025-11-17

**Authors:** Pattara Saardarwut, Kakanang Piyarungsri, Nawin Manachai, Sahatchai Tangtrongsup

**Affiliations:** 1 Master of Science Program in Veterinary Science, Faculty of Veterinary Medicine, Chiang Mai University, Chiang Mai 50100, Thailand; pattara_s@cmu.ac.th; 2 Faculty of Veterinary Medicine, Chiang Mai University, Chiang Mai 50100, Thailand; kakanangjp@gmail.com (K.P.); nawin.m@cmu.ac.th (N.M.); 3 Research Center of Producing and Development of Products and Innovations for Animal Health and Production, Faculty of Veterinary Medicine, Chiang Mai University, Chiang Mai 50100, Thailand; 4 Elephant, Wildlife, and Companion Animals Research Group, Faculty of Veterinary Medicine, Chiang Mai University, Chiang Mai 50100, Thailand

**Keywords:** dog, azotemia, risk factors, epidemiology, Chiang Mai, Thailand

## Abstract

Kidney disorders in dogs are frequently observed but remain underexplored in Chiang Mai, Thailand. This retrospective study aimed to estimate the prevalence and identify risk factors associated with azotemia in dogs presented to the Chiang Mai University Small Animal Veterinary Teaching Hospital between May 2017 and December 2021. Medical and laboratory records from 16,146 dogs were reviewed, and demographic, lifestyle, hematologic, and biochemical factors were analyzed using univariable and multivariable logistic regression to determine associations with azotemia. Overall, 3505 dogs (21.7%) were classified as azotemic, of which 43.5% had acute kidney injury, 5.9% had chronic kidney disease, and 50.6% were undetermined due to incomplete records. During winter, being mixed-breed, of older age, male sex, being fed with homemade and mixed diets, and drinking tap or mixed water sources were significantly associated with increased risk of azotemia (*p* < 0.05), whereas living outdoors or indoor-outdoor reduced the risk. Affected dogs typically showed elevated urea and creatinine concentrations, anemia, and abnormal white blood cell profiles, indicating systemic inflammation or dehydration. Concurrent diseases that were significantly associated with azotemia included pancreatitis, trauma, urinary tract injury, pyometra, and cardiovascular disorders. These findings indicate that canine azotemia in this region arises from multifactorial causes, emphasizing the need for early screening, balanced nutrition, access to clean water, and timely management of concurrent diseases are essential to improve kidney health and clinical outcomes in dogs.

## Simple Summary

Kidney problems are common in dogs and may be influenced by age, diet, water quality, lifestyle, and other diseases. This study reviewed medical records of more than 16,000 dogs visiting the Chiang Mai University Small Animal Veterinary Teaching Hospital between 2017 and 2021 to determine the prevalence of kidney problems and the risk factors. Approximately one in five dogs exhibited reduced kidney function, with cases occurring most frequently during the winter season. Most had acute kidney injury, while fewer had chronic kidney disease. Old and male dogs, mixed-breed dogs, dogs eating homemade or mixed food, dogs living partly indoors and partly outdoors, and dogs drinking tap or mixed types of water were significantly associated with kidney problems. Azotemic dogs also showed blood changes, including elevated urea and creatinine concentrations, anemia, and white blood cell abnormalities, indicating systemic stress and inflammation. Common concurrent diseases, including pancreatitis, trauma, urinary tract injuries, pyometra, and heart disorders, were significantly linked to the development of azotemia. In summary, kidney problems were multifactorial and linked to age, sex, lifestyle, diet, and season. These findings emphasize the need for routine kidney screening and preventive care to improve dogs’ kidney health and overall well-being in tropical regions like Chiang Mai, Thailand.

## 1. Introduction

Azotemia, characterized by an increased concentration of nonprotein nitrogenous compounds such as blood urea nitrogen (BUN) and serum creatinine (SCr), is a key biochemical indicator of impaired kidney function in dogs [1,2]. Azotemia was defined as BUN ≥ 28 mg/dL and/or SCr > 1.7 mg/dL. Clinically, azotemic dogs may present with nonspecific signs such as lethargy, anorexia, vomiting, polyuria, polydipsia, and dehydration, reflecting accumulation of metabolic waste and altered fluid balance. Depending on the underlying cause, azotemia can be classified as prerenal, resulting from a simultaneous decrease in glomerular filtration rate induced by volume depletion, vascular collapse, thrombotic diseases, and shock. The occurrence of renal azotemia is predisposed by nephrons’ destruction by toxins, infectious agents, inflammatory, ischemic, or neoplastic processes. In comparison, postrenal azotemia plays a different role due to obstruction of the urinary tract. It impairs the elimination of waste products in urine, leading to fluid, electrolyte, and acid-base imbalances. Nonetheless, intrinsic renal parenchyma injury can be affected by pre- and postrenal azotemia [2,3].

Azotemia can be acute and profound or chronic and mild/progressive [3,4,5,6]. Acute kidney injury (AKI) is defined as a sudden and potentially reversible decline in renal function. In contrast, chronic kidney disease (CKD) involves progressive and irreversible structural or functional loss over time [3,7,8,9]. Both conditions share overlapping clinical and biochemical features, posing diagnostic and management challenges, particularly in general practice settings [10]. However, climate, diet, housing conditions, and water quality may also influence renal health in dogs. Seasonal temperature variation and region-specific infectious diseases further complicate the clinical landscape, suggesting potential geographical differences in risk patterns [10,11].

To date, no comprehensive study has evaluated the prevalence and associated risk factors of azotemia among canine patients in Chiang Mai, Thailand. Addressing this gap is essential for understanding the regional epidemiology of kidney disease and for developing targeted prevention and management strategies suited to the tropical environment.

Despite the clinical importance of kidney disorders, large-scale epidemiological data on canine azotemia in this region are scarce. Previous studies have primarily focused on small case series or specific etiologies, such as leptospirosis, toxins, or pyometra [8,12,13,14,15]. Reports on concurrent systemic illnesses such as pancreatitis, trauma, urinary tract injury, cardiovascular disease, and reproductive infections have been associated with renal impairment through multifactorial mechanisms, including hypoperfusion, inflammation, and toxin exposure [6,8,14,16]. Still, these relationships have not been thoroughly investigated. Moreover, although international guidelines, such as those from the International Renal Interest Society (IRIS) [17,18], have standardized the grading and staging of AKI and CKD, their application and epidemiological reporting in veterinary patients, particularly in Chiang Mai, Thailand, remain limited.

Therefore, this study aimed to estimate the prevalence of, classify, and assess risk factors associated with azotemia in dogs presented to the Chiang Mai University Small Animal Veterinary Teaching Hospital in Chiang Mai, Thailand, between 2017 and 2021.

## 2. Materials and Methods

### 2.1. Study Design

A retrospective study was conducted using medical records from the Small Animal Veterinary Teaching Hospital, Faculty of Veterinary Medicine, Chiang Mai University. Data from dogs presented between May 2017 and December 2021 were reviewed. Dogs were classified as azotemic if blood urea nitrogen (BUN) was ≥ 28 mg/dL and/or serum creatinine (SCr) > 1.7 mg/dL [19]. Non-azotemic controls were randomly selected from dogs examined during the same period. Cases and controls with incomplete records or missing biochemical data were excluded. Azotemic dogs were classified according to the International Renal Interest Society (IRIS) guidelines as AKI and CKD [17]. An undetermined group referred to cases with azotemic blood results but lacking sufficient diagnostic data, based on clinical history, physical examination, urinalysis, and ultrasonography.

### 2.2. Data Collection

Demographic and clinical data were obtained from hospital records, and telephone interviews with dog owners were conducted when demographic information was incomplete. Recorded variables included breed, age, sex, diet type (commercial, homemade, mixed), lifestyle (indoor, outdoor, mixed), source of drinking water (bottled, tap, mixed), and concurrent diseases (e.g., pancreatitis, trauma, urinary tract injury, pyometra, cardiovascular disease, leptospirosis, and toxin exposure). Clinical data included hematological and biochemical parameters.

### 2.3. Statistical Analysis

Descriptive statistics were expressed as mean ± SD or percentage with 95% confidence interval (95% CI) as per the type of collected data. Risk factors (breed, sex, age, hematological findings, and concurrent diseases) and azotemia were assessed using univariable and multivariable logistic regression analyses. Variables associated with azotemia from univariable logistic regression analysis (*p* < 0.1) were included in the multivariable logistic regression analysis. The backward stepwise elimination procedure was performed to eliminate the least significant variables. Variables retained in the model were based on the likelihood ratio χ^2^ statistic (*p* < 0.05). Fisher’s exact test was used to assess the association of azotemia and risk factors when the logistic regression was not applicable. All analyses were performed using Stata 16.1 (StataCorp, College Station, TX, USA), with statistical significance set at *p* < 0.05.

## 3. Results

**Figure 1:**
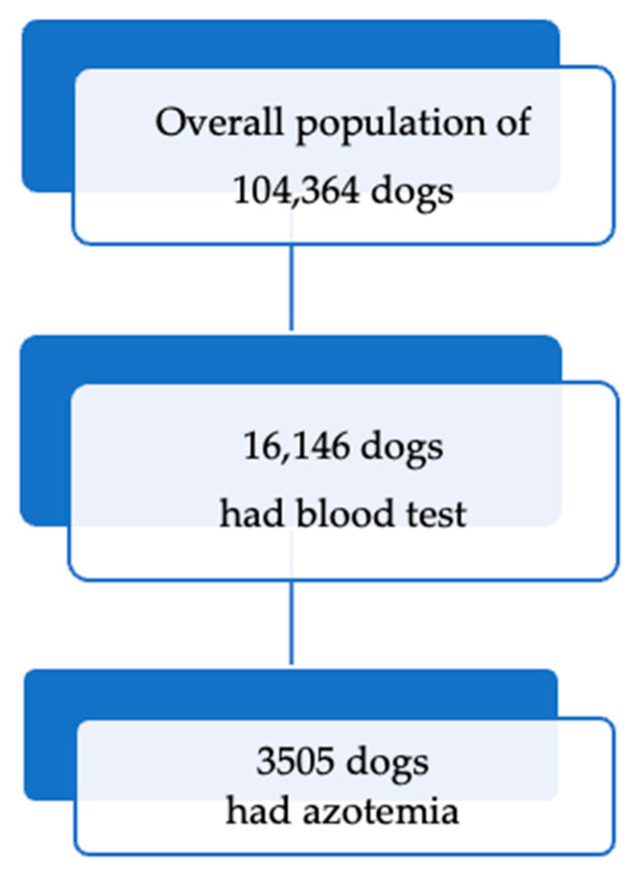
Distribution of total dogs, tested dogs, and azotemic cases.

**Figure 2:**
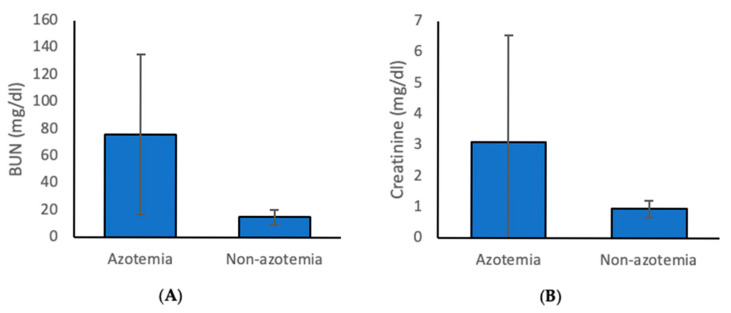
Comparison of (**A**) blood urea nitrogen (BUN) and (**B**) serum creatinine (SCr) levels between azotemic and non-azotemic dogs.

From May 2017 to December 2021, a total of 104,364 dogs visited the Chiang Mai University Small Animal Veterinary Teaching Hospital, and 16,146 dogs had blood chemistry tests. Of these, 3505 dogs were identified as azotemic (Figure 1). On average, BUN and Creatinine levels in azotemic dogs were 76.08 ± 59.29 mg/dl and 3.1 ± 3.46 mg/dl, respectively (Figure 2). The breeds of azotemic dogs that were most commonly identified were mixed breed (1608 dogs, 45.9%), Pomeranian (333 dogs, 9.5%), Shih Tzu (299 dogs, 8.5%), Poodle (291 dogs, 8.3%), and Chihuahua (213 dogs, 6.1%) (Table 1). There were 1846 (52.8%) male and 1653 (47.2%) female dogs with azotemia.

### 3.1. Prevalence and Classification of Azotemia

The overall prevalence of azotemia in dogs was 3.4% (3505/104,364; 95% CI: 3.3–3.5) based on total hospital visits, and 21.7% (3505/16,145; 95% CI: 21.1–22.4) among dogs that underwent laboratory testing. Of azotemic dogs, 43.48% (1524/3505) were diagnosed with AKI, 5.93% (208/3505) with CKD, and 50.58% (1773/3505) were classified as undetermined due to incomplete diagnostic information. Within AKI cases, IRIS grade 3 was most common (29.5%), followed by grades 2 (23.1%), 4 (22.8%), 1 (12.9%), and 5 (11.7%). In contrast, most CKD dogs were classified as stage 2 (46.2%), followed by stage 3 (27.4%) and stage 1 (26.4%), with no dogs identified at stage 4.

### 3.2. Risk Associated with Azotemia in Dogs

Univariable logistic regression analysis was used to estimate the risk associated with azotemia in dogs (Table 2). Dogs were more likely to be azotemic in winter (OR: 1.70, 95% CI: 1.47–1.97) and less likely in rainy (OR: 0.83, 95% CI: 0.73–0.95) than in summer. Mixed-breed dogs were more likely to develop azotemia than purebred (OR: 1.32, 95% CI: 1.18–1.48). Dogs younger than 6 months (OR: 1.42, 95% CI: 1.05–1.93), dogs aged >1–7 years (OR: 1.49, 95% CI: 1.20–1.85), >7–10 years (OR: 2.54, 95% CI: 2.02–3.21), and >10 years (OR: 3.57, 95% CI: 2.84–4.49) were more likely to become azotemic than dogs of 6 months–1 years old. Male dogs were also at slightly higher risk than females (OR: 1.14, 95% CI: 1.02–1.27). Dogs fed with homemade (OR: 1.72, 95% CI: 1.49–1.98) and mixed diets (OR: 1.58, 95% CI: 1.39–1.80) had an increased risk compared with dogs receiving commercial diets. Regarding lifestyle, mixed (indoor–outdoor) dogs exhibited a lower risk (OR: 0.79, 95% CI: 0.69–0.91) than indoor dogs. Dogs drinking tap water (OR: 1.15, 95% CI: 1.03–1.30) and mixed water sources (OR: 27.06, 95% CI: 3.70–197.55) were at greater risk than those drinking clean or bottled water.

Several concurrent conditions were significantly associated with azotemia. Dogs with trauma had a markedly higher risk of azotemia (OR: 13.68, 95% CI: 7.98–23.44), as did those with diarrhea (OR = 4.62, 95% CI: 2.46–8.67). Other concurrent illnesses associated with azotemia were cardiovascular diseases (OR: 3.75, 95% CI: 2.13–6.61) and anemia (OR: 4.07, 95% CI: 3.54–4.70). Other frequently observed conditions included pancreatitis, urinary bladder or urethral injury, and pyometra, all found exclusively among azotemic dogs but not valid for univariable analysis (*p* < 0.001). Hematologic alterations were associated with azotemic dogs. Both leukocytosis (OR: 2.65, 95% CI: 2.35–2.99) and leukopenia (OR: 2.06, 95% CI: 1.50–2.84) were significantly associated with azotemia. Similarly, neutropenia (OR = 3.09, 95% CI: 2.29–4.18) and neutrophilia (OR = 2.90, 95% CI: 2.57–3.27) were associated with renal dysfunction, suggesting that systemic inflammation, infection, and sepsis-associated renal impairment play a pivotal role in the pathogenesis of azotemia in dogs. Of all azotemic dogs, 55 were tested for leptospirosis by the Polymerase chain reaction (PCR) method, and 40% were positive. All positive results were reported in AKI dogs. However, due to the limited number of tested samples, these findings were not included in the statistical analysis.

The variables remaining in the multivariable logistic regression model that were associated with an increased risk were dogs age > 1 year, being male, fed with a homemade or mixed diet, drinking mixed types of water, patients in winter, anemic, leukocytosis, neutropenia, neutrophilia, trauma, having diarrhea, and cardiovascular diseases (Table 3).

## 4. Discussion

This study provides the first large-scale hospital-based epidemiological analysis of azotemia in dogs in Chiang Mai, Thailand, revealing that approximately one-fifth (21.7%) of dogs undergoing blood testing exhibited azotemia. Older and male dogs, particularly mixed breeds, as well as those fed with homemade or mixed diets, living in both indoor and outdoor environments, and consuming tap or mixed water sources, were significantly associated with an increased risk of azotemia. Azotemic dogs exhibited hematological and biochemical alterations, including elevated urea and creatinine concentrations, anemia, and leukocyte abnormalities, reflecting systemic stress and inflammation. Furthermore, concurrent disorders such as pancreatitis, trauma, urinary tract injuries, pyometra, and cardiovascular diseases were significantly linked to the development of azotemia. Leptospirosis was found positive among AKI dogs. However, this finding was not included in the statistical analysis due to the limited number of tested samples.

The observed prevalence was notably higher than that reported in previous studies from temperate regions such as the United Kingdom, where azotemia, AKI, and CKD rates typically range between 0.05–11% depending on study design and population [10,20,21,22,23]. This discrepancy may be attributable to regional differences in environmental exposure, infectious disease burden, and owner management practices. The findings highlight that climatic and lifestyle factors specific to Chiang Mai, Thailand, play a crucial role in canine renal health.

The predominance of AKI (43.5%) over CKD (5.9%) aligns with previous reports suggesting that acute renal insults are more frequently encountered in tropical clinical settings, where dehydration, heat stress, and infectious etiologies such as leptospirosis are common [3,5,6,8,11,14,23,24,25,26,27]. The large proportion of “undetermined” cases (50.6%) likely reflects diagnostic limitations inherent to retrospective studies. Nevertheless, this unidentified group may have included both early or overlapping stages of AKI and CKD that were not clinically confirmed. The absence of stage 4 cases and the limited availability of IRIS sub-staging data—particularly proteinuria and blood pressure measurements—further highlight the challenges of accurately classifying CKD severity in retrospective studies. Future studies integrating complete diagnostic criteria could thus refine prevalence estimates and strengthen causal inference for azotemia in dogs.

Seasonal analysis showed a significantly higher prevalence of azotemia during the cool season, consistent with patterns observed in other tropical studies where climatic fluctuations affect hydration status and disease transmission [13,28,29,30,31,32]. In addition, infectious diseases such as leptospirosis tend to surge following periods of rainfall, and clinical cases may present weeks later during cooler months [28,29]. This temporal link between rainfall, environmental exposure, and renal insult supports the hypothesis that climatic conditions are an indirect but significant contributor to azotemia in tropical environments.

Age, sex, and breed were important determinants of azotemia. Our study demonstrated that dogs older than 10 years had the highest risk of developing azotemia compared to other age groups. Older dogs showed a progressive increase in risk, consistent with renal senescence and cumulative exposure to nephrotoxic factors described in prior studies [3,8,33]. In the present study, male dogs were identified as a risk factor for the development of azotemia. Males were more frequently affected, possibly due to hormonal influences on renal hemodynamics or behavioral patterns predisposing to dehydration and environmental toxin exposure [22,34,35].

Mixed-breed dogs showed a significant association with azotemia in the univariable logistic regression analysis; however, breed was not retained in the multivariable model, suggesting that it acted as a confounding rather than an independent factor. This relationship is likely explained by correlations between breed and other variables—such as age, diet, housing conditions, or owner management style—that more directly influence renal health.

Owner management factors, including homemade or mixed diets, mixed water sources, and outdoor or mixed (indoor–outdoor) lifestyles, were significantly correlated with azotemia. These findings suggest that environmental quality and feeding practices—particularly unbalanced homemade diets or exposure to contaminated water—may contribute to renal stress and compromise renal function. Interestingly, the lower odds of azotemia observed in dogs with outdoor or indoor–outdoor lifestyles in the present study contrast with previous reports linking outdoor exposure to a higher risk of AKI due to environmental factors such as leptospirosis and heat-related dehydration [28,29]. This discrepancy may reflect regional differences in environmental management, owner care practices, or the relatively controlled outdoor conditions of companion animals in urban Chiang Mai, where dogs with regular outdoor access may also benefit from better exercise, hydration, and owner supervision.

Consistent with the definition of azotemia, affected dogs showed marked increases in blood urea nitrogen and creatinine, along with anemia and leukocytic abnormalities. These alterations are compatible with systemic inflammation, dehydration, or secondary effects of renal impairment [5,8,36]. Electrolyte disturbances such as hyponatremia and hyperphosphatemia were frequently observed, supporting impaired renal excretory function. Similar findings have been reported in previous studies, in which azotemic dogs commonly exhibited elevated serum creatinine, blood urea nitrogen, phosphorus, and creatine kinase (CK) as well as anemia [8,37]. Hypochloremia, hyperkalemia, hypocalcemia, hyponatremia, and metabolic acidosis were frequently reported in AKI dogs [38,39,40]. The presence of both hematologic and biochemical abnormalities reinforces the multifactorial nature of renal compromise, where systemic disease, hemodynamic instability, and intrinsic renal injury coexist.

Pancreatitis, trauma, urinary tract injury, pyometra, and cardiovascular disorders were the most common concurrent diseases associated with azotemia. These conditions likely contribute to renal dysfunction via mechanisms such as hypoperfusion, inflammatory mediator release, and secondary infection.

Infectious diseases that have been reported to be associated with the development of acute kidney injury (AKI) and chronic kidney disease (CKD) in tropical areas include leptospirosis, ehrlichiosis, babesiosis, and anaplasmosis, which can induce renal damage through direct pathogen invasion, immune-mediated injury, or systemic inflammation [41,42,43]. In this study, 55 azotemic dogs were tested for *Leptospira* spp. infection with 40% PCR-positive results, either from blood or urine samples. This finding supports prior reports identifying *Leptospira* spp. as a leading cause of acute renal failure in dogs [12,13,44]. Several serovars have been reported across regions, reflecting high antigenic diversity. In Chiang Mai, serogroups *Batavia*, *Canicola*, *Australis*, and *Icterohaemorrhagiae* predominate [45], whereas rural surveys have identified *Ranarum*, *Saigon*, *Bratislava*, *Copenhageni*, and others [46]. Local isolates from asymptomatic dogs include *L. interrorgans* serovars *Bataviae* and *Grippotyphosa* [47]. Another study identified *Sejroe* (4.4%), *Icterohaemorrhagiae* (3.7%), *Bataviae* (2.9%), and *Canicola* (2.6%) as predominant serogroups among infected dogs, with high MAT titers and positive urine PCR results [12]. These data collectively indicate that a wide range of *Leptospira* serovars circulate in Thailand, contributing to the ongoing risk of renal involvement in canine populations.

Currently available leptospiral vaccines in Thailand are limited to bivalent, trivalent, or quadrivalent formulations, depending on the product and manufacturer. The bivalent vaccine comprises only *Icterohaemorrhagiae* and *Canicola*. The trivalent formulation includes *Icterohaemorrhagiae*, *Canicola*, and *Grippotyphosa*. The quadrivalent vaccine contains four *Leptospira interrogans* serovars—*Icterohaemorrhagiae*, *Canicola*, *Grippotyphosa*, and *Pomona* [47]. However, these vaccine formulations do not provide full coverage against locally prevalent serovars such as *Bataviae* and other emerging serovars. Therefore, it cannot be conclusively determined that PCR- or MAT-positive cases in vaccinated dogs represent vaccine failure; instead, they reflect the limited cross-protection of current formulations. Vaccination may mitigate disease severity but cannot entirely prevent bacterial shedding or transmission [45]. These findings underscore the need for continuous regional surveillance and the updated vaccine formulations incorporating locally prevalent serovars to enhance preventive efficacy in Thailand.

The results collectively demonstrate that canine azotemia in tropical regions such as Chiang Mai, Thailand, is influenced by an intricate interplay of demographic, environmental, and systemic factors. Clinically, these findings underscore the necessity for veterinarians to integrate lifestyle and seasonal risk assessment into renal disease prevention programs. Routine monitoring of renal function—particularly in older, male, and purebred dogs—together with improved owner education on balanced diets and safe water sources, could significantly reduce disease burden.

This study has certain limitations. The estimated overall prevalence of azotemia may not reflect the true prevalence due to the retrospective nature of the study, which focused on canine patients with azotemia. In addition, the Chiang Mai University Small Animal Veterinary Teaching Hospital is a secondary and/or tertiary facility; manageable azotemic AKI or CKD dogs could have been treated elsewhere prior to coming to the hospital, which may not reflect the population of dogs in Chiang Mai at large. Also, incomplete medical records and financial constraints could have affected the overall clarity of the diagnosis and data, potentially leading to information bias.

Future studies should adopt a prospective design incorporating standardized diagnostic follow-up, renal biomarkers (e.g., SDMA, NGAL) [9,48], and molecular surveillance of infectious agents such as *Leptospira* and tick-borne pathogens [31,42,49]. Expanding data integration with regional climate and water-quality indices may further elucidate the environmental drivers of canine renal disease.

## 5. Conclusions

This study provides the first large-scale epidemiological overview of canine azotemia in Chiang Mai, Thailand, revealing that approximately one-fifth of dogs exhibited biochemical evidence of impaired renal function. Acute kidney injury was the predominant form, followed by chronic kidney disease. Canine azotemia results from multifactorial causes, especially pancreatitis, trauma, urinary tract injury, pyometra, and cardiovascular disorders. Aging, male sex, homemade diets, mixed water sources, and winter season are risk factors for azotemia in dogs. In contrast, a mixed lifestyle, an outdoor lifestyle, and the rainy season serve as protective factors for dogs with azotemia. These findings highlight the need for early renal screening in the aged population, improved dietary and water hygiene practices, and timely management of concurrent diseases. Future studies should incorporate standardized diagnostic protocols and manage the causes of azotemia in dogs.

## Figures and Tables

**Table 1:** Breed distribution of dogs with azotemia presenting at Chiang Mai University Small Animal Veterinary Teaching Hospital during 2017–2021.

Breed (*n* = 3505)	Number (%)
Mixed breed	1608 (45.9)
Pomeranian	333 (9.5)
Shih tzu	299 (8.5)
Poodle	291 (8.3)
Chihuahua	213 (6.1)
Golden retriever	105 (3)
Siberian husky	103 (2.9)
Bangkaew	76 (2.2)
Labrador retriever	66 (1.9)
Pug	43 (1.2)
Miniature Pincher	40 (1.1)
French Bulldog	37 (1.1)

*Note:* All breeds representing ≥1.0 per cent of the group are included.

**Table 2:** Univariable logistic regression of risk factors associated with azotemia in dogs in Chiang Mai, Thailand.

Variables	Azotemia (*N*, %)	Odds Ratio (95%CI)	*p*-Value
**Season**			
Summer	850/1389 (61.2)	*Reference*	
Rainy	1290/2272 (56.8)	0.83 (0.73–0.95)	**0.009**
Winter	1365/1874 (72.8)	1.70 (1.47–1.97)	**<0.001**
**Breed**			
Mixed breed	1608/2401 (67.0)	1.32 (1.18–1.48)	**<0.001**
Pure breed	1895/3130 (60.5)	*Reference*	
**Age**			
<6 months	161/292 (55.1)	1.42 (1.05–1.93)	**0.023**
6 months–1 year	184/397 (46.4)	*Reference*	
>1–7 years	1230/2186 (56.3)	1.49 (1.20–1.85)	**<0.001**
>7–10 years	798/1161 (68.7)	2.54 (2.02–3.21)	**<0.001**
>10 years	1132/1499 (75.5)	3.57 (2.84–4.49)	**<0.001**
**Sex**			
Female	1653/2678 (61.7)	*Reference*	
Male	1846/2849 (64.8)	1.14 (1.02–1.27)	**0.018**
**Types of food**			
Commercial diet	1188/2119 (56.1)	*Reference*	
Homemade	991/1443 (68.7)	1.72 (1.49–1.98)	**<0.001**
Milk	5/5 (100)	N/A	
Mixed	1304/1949 (66.9)	1.58 (1.39–1.80)	**<0.001**
Raw	2/3 (66.7)	1.56 (0.14–17.31)	0.714
**Lifestyle**			
Indoor	1186/1830 (64.8)	*Reference*	
Outdoor	1360/2091 (65.0)	1.01 (0.89–1.15)	0.879
Mixed lifestyle	945/1595 (59.3)	0.79 (0.69–0.91)	**0.001**
**Water source**			
Drinking water	1011/1731 (58.4)	*Reference*	
Milk	4/5 (80.0)	2.85 (0.32–25.54)	0.35
Mixed water	38/39 (97.4)	27.06 (3.70–197.55)	**0.001**
Tap water	2111/3413 (61.9)	1.15 (1.03–1.30)	**0.017**
Natural water	3/3 (100)	N/A	
**Trauma**			
Yes	304/318 (95.6)	13.68 (7.98–23.44)	**<0.001**
No	3201/5217 (61.4)	*Reference*	
**Diarrhea**			
Yes	86/97 (86.7)	4.62 (2.46–8.67)	**<0.001**
No	3419/5438 (62.9)	*Reference*	
**Blood parasite**			
Yes	98/144 (68.1)	1.24 (0.87–1.77)	0.233
No	3407/5391 (63.2)	*Reference*	
**Kidney stone**			
Yes	1/1 (100)	N/A	<0.001 *
No	3504/5534 (63.3)		
**IMHA**			
Yes	2/4 (50)	0.58 (0.08–4.11)	0.585
No	3503/5531 (63.3)	*Reference*	
**Pancreatitis**			
Yes	38/38 (100)	N/A	<0.001 *
No	3467/5497 (63.1)		
**UB/Urethra injury**			
Yes	42/42 (100)	N/A	<0.001 *
No	3463/5493 (63.0)		
**UTIs**			
Yes	23/23 (100)	N/A	<0.001 *
No	3482/5512 (63.2)		
**Pyometra**			
Yes	180/180 (100)	N/A	<0.001 *
No	3325/5355 (62.1)		
**Respiratory**			
Yes	50/68 (73.5)	1.62 (0.94–2.78)	0.082
No	3455/5467 (63.2)	*Reference*	
**CVS**			
Yes	89/103 (86.4)	3.75 (2.13–6.61)	**<0.001**
No	3416/5432 (62.9)	*Reference*	
**Toxin**			
Yes	12/12 (100)	N/A	0.005 *
No	3493/5523 (63.2)		
**Anemia**			
Yes	1424/1716 (83.0)	4.07 (3.54–4.70)	**<0.001**
No	2081/3819 (54.5)	*Reference*	
**WBCs**			
Normal	1748/3211 (54.4)	*Reference*	
Leukocytosis	1619/2130 (76.0)	2.65 (2.35–2.99)	**<0.001**
Leukopenia	138/194 (71.1)	2.06 (1.50–2.84)	**<0.001**
**Neutrophils**			
Normal (3020)	1585/3020 (52.5)	*Reference*	
Neutropenia (256)	198/256 (77.3)	3.09 (2.29–4.18)	**<0.001**
Neutrophilia (2259)	1722/2259 (76.2)	2.90 (2.57–3.27)	**<0.001**

Abbreviation; IMHA, Immune mediated hemolytic anemia, UB; Urinary bladder, UTIs; Urinary tract infection, CVS; Cardiovascular system, N/A; not applicable; * The *p*-value from Fisher’s exact tests; Bold values indicate *p* < 0.05.

**Table 3:** Multivariable logistic regression of risk factors associated with azotemia in dogs in Chiang Mai, Thailand.

Variables	Odds Ratio (95%CI)	*p*-Value
>1–7 years	1.77 (1.36–2.29)	<0.001
>7–10 years	2.77 (2.10–3.67)	<0.001
>10 years	4.28 (3.25–5.64)	<0.001
Male	1.18 (1.04–1.34)	0.010
Homemade	1.41 (1.18–1.69)	<0.001
Mixed types of food	1.36 (1.16–1.60)	<0.001
Outdoor	0.70 (0.57–0.87)	0.001
Indoor-outdoor	0.65 (0.54–0.78)	<0.001
Mixed type of water	24.00 (3.19–180.67)	0.001
Rainy	0.67 (0.57–0.78)	<0.001
Winter	1.65 (1.39–1.95)	<0.001
Anemia	3.85 (3.29–4.52)	<0.001
Leukocytosis	1.30 (1.04–1.63)	0.019
Neutropenia	2.70 (1.86–3.95)	<0.001
Neutrophilia	1.94 (1.56–2.41)	<0.001
Trauma	17.95 (10.32–31.21)	<0.001
Diarrhea	5.93 (3.05–11.51)	<0.001
Cardiovascular diseases	4.00 (2.18–7.35)	<0.001

## Data Availability

Data is contained within the article.

## Abbreviations

AKI	Acute kidney injury
BUN	Blood urea nitrogen
CI	Confidence intervals
CKD	Chronic kidney disease
CVS	Cardiovascular disease
IRIS	International Renal Interest Society
NGAL	Neutrophil gelatinase-associated lipocalin
OR	Odds ratios
PCR	Polymerase chain reaction
SCr	Serum creatinine
SDMA	Symmetric dimethylarginine
UK	United Kingdom
USA	United States of America
